# Decision‐making trends in therapeutic interventions for multiple system atrophy: a 24‐year retrospective study

**DOI:** 10.1002/mdc3.70000

**Published:** 2025-02-14

**Authors:** Katsuya Nishida, Kento Sakashita, Naonobu Futamura

**Affiliations:** ^1^ Department of Neurology National Hospital Organization Hyogo Chuo National Hospital Hyogo Japan

**Keywords:** multiple system atrophy, tracheostomy invasive ventilation, tracheostomy, enteral nutrition, decision‐making

## Abstract

**Background:**

Managing multiple system atrophy (MSA) is challenging. While invasive interventions for amyotrophic lateral sclerosis are well‐studied, those for MSA remain less explored.

**Objectives:**

To explore factors influencing treatment choices and trends in advanced‐stage MSA.

**Methods:**

A retrospective cohort study analyzed 128 MSA patients at Hyogo Chuo National Hospital, Japan, from 2000 to 2024, focusing on treatment period and age at onset.

**Results:**

Tracheostomy invasive ventilation (TIV) decreased after 2014 (26.9% vs. 9.2%; *P* = 0.023). TIV‐treated patients remained similarly young before and after 2014 (age at onset 52.7 vs. 54.5 years; *P* = 0.659) and tracheostomy was chosen by younger patients after 2014 (58.3 vs. 51.5 years; *P* < 0.001). Conversely, enteral nutrition increased in older patients (57.4 vs. 62.9 years; *P* = 0.011).

**Conclusions:**

In Japanese MSA, preferences for invasive treatments shifted, with younger patients favoring TIV and tracheostomy, while older patients preferred less invasive options, emphasizing personalized care.

Neurodegenerative diseases present challenges for patients, families, and healthcare providers, requiring thoughtful consideration of interventions. In Japan, invasive procedures like tracheostomy invasive ventilation (TIV) are more common than in other countries.^1^ In contrast to amyotrophic lateral sclerosis (ALS), research on invasive interventions for multiple system atrophy (MSA) is limited.

MSA is a rare, adult‐onset neurodegenerative disorder affecting the autonomic nervous system and motor function.^2^ It is characterized by abnormal autonomic symptoms, parkinsonism, and cerebellar ataxia, manifesting as MSA‐P (predominant parkinsonism) and MSA‐C (predominant cerebellar features).^3^ In advanced stages, interventions such as enteral nutrition, tracheostomy alone, and TIV may become necessary.^4^

This study examines factors influencing invasive interventions in MSA, where treatment decisions, like in ALS, are complex. Trends in invasive procedures reflect changing patient preferences, impacting clinical practices and policies. Various factors, including cultural influences and healthcare systems, may affect the frequency of these interventions. We aimed to assess changes in treatment decisions for MSA patients over 24 years to provide insights for optimizing care strategies.

## Methods

### Study overview

This retrospective cohort study analyzed medical records of advanced‐stage MSA patients treated at the National Hospital Organization Hyogo Chuo National Hospital, Japan, between January 1, 2000, and January 1, 2024. The cohorts in our study were defined as (1) patients who opted for invasive treatments (eg, TIV, tracheostomy, or enteral nutrition) versus those who did not, and (2) patients treated before 2014 versus those treated after 2014.

### Inclusion criteria

We reviewed records of patients diagnosed with MSA using the Gilman diagnostic criteria, which were the standard during most of the study period.^5^ The study included both patients who opted for invasive interventions (such as TIV, tracheostomy, or enteral nutrition) and those who did not undergo invasive treatments, providing a comprehensive analysis of treatment preferences and outcomes. Non‐invasive ventilation was excluded due to its less invasive nature. Comprehensive medical records were available for all patients, clearly documenting whether invasive treatments were performed or explicitly refused by the patients or their families. Cases with incomplete or unclear records, such as those missing key medical history, diagnostic information, or documentation of patient or family intent, were excluded; however, no such cases were identified in this study. All patients were followed up until death or the end of the observation period, minimizing the risk of incomplete data due to treatment at other facilities. Additionally, patients who had not progressed to a stage requiring invasive procedures by the end of the observation period were excluded from the analysis.

### Data collection and analysis

We collected data on sex, clinical phenotype (MSA‐C or MSA‐P), age at onset, age at intervention, time to treatment milestone, hospitalization duration, and overall survival. Disease onset was defined as the first reported symptom, whether autonomic or motor. We calculated the proportions of patients opting for each intervention, dividing the investigation period into two groups: before and after 2014.

### Specific intervention criteria

Tracheostomy was performed based on stridor severity, with further laryngofiberscopy by an otolaryngologist. TIV was initiated when hypoxemia or hypercapnia was confirmed by oxygen saturation monitoring or blood gas analysis. Decision‐making regarding enteral nutrition involved a multidisciplinary team of neurologists, gastroenterologists, speech‐language‐hearing therapists, and dietitians. If a patient declined invasive treatment, supportive care was continued, reflecting a patient‐centered approach prioritizing individual preferences and autonomy.

### Ethical considerations

The study was approved by the institutional review board of Hyogo Chuo National Hospital (approval number: 20–05). An opt‐out consent model was used to respect patient autonomy and confidentiality.

### Statistical analysis

Summary statistics were calculated as means (and standard deviations) for continuous variables and as frequencies (and percentages) for categorical variables. Normality of continuous variables was confirmed using the Shapiro–Wilk test. Continuous variables were compared using the *t*‐test or Mann–Whitney U test, depending on the normality. Categorical variables were compared using either the chi‐square test or Fisher's exact test, depending on sample size and expected frequencies. Statistical analyses were performed using EZR, version 1.61, a graphical interface for R version 4.2.2 (The R Foundation for Statistical Computing, Vienna, Austria)^6^; significance was indicated by a *P*‐value of <0.05.

## Results

Among 128 patients with definite or probable MSA, 112 (87.5%) died, and 12 (9.4%) had a pathological diagnosis. MSA‐C was present in 85 cases (66.4%), and MSA‐P in 43 (33.6%). Of the patients, 66 (51.6%) were male and 64 (48.4%) were female. The average age at onset was 61.4 years, and median survival after onset was 8.0 years (Table 1).

**TABLE 1:** Clinical characteristics of the study population with multiple system atrophy

Characteristic	Overall (*n* = 128)	MSA‐C (*n* = 85)	MSA‐P (*n* = 43)	*P* value
Sex, *n* (%)				
Female	62 (48.4)	37 (28.9)	25 (19.5)	0.169
Male	66 (51.6)	48 (37.5)	18 (14.0)	
Death, *n* (%)	112 (87.5)	72 (56.3)	40 (31.3)	0.289
Diagnostic certainty, *n* (%)				
Definite	12 (9.4)	8 (6.3)	4 (3.1)	1.000
Probable	116 (90.6)	77 (60.2)	38 (29.7)	1.000
Age at onset, y	61.4 (9.5)	60.2 (8.6)	63.7 (10.9)	0.045
Survival time, y	8.0 (5.7–12.6)	8.6 (6.0–13.1)	7.7 (5.7–11.6)	0.656
Hospitalization, y	2.1 (0.5–5.1)	2.5 (0.43–5.3)	1.7 (0.5–4.3)	0.400
Intervention, *n* (%)				
Enteral nutrition	84 (65.6)	49 (57.6)	35 (81.4)	0.013
Gastrostomy tube	65 (50.8)	39 (30.5)	26 (20.3)	0.758
Nasogastric tube	19 (14.8)	10 (7.8)	9 (7.0)	
Tracheostomy alone	55 (43.0)	39 (45.9)	16 (37.2)	0.455
Tracheostomy invasive ventilation	20 (15.6)	15 (17.6)	5 (11.6)	0.530

Abbreviations: MSA, multiple system atrophy; MSA‐C, MSA‐cerebellar type; MSA‐P, MSA‐parkinsonian type; y, years (mean/standard deviation or median/interquartile range).

TIV was chosen for 20 patients (15.6%), and 97 patients (75.8%) died without TIV (Table 2). These patients were significantly younger at disease onset than those not treated with TIV (mean age, 53.2 vs. 63.6 years; *P* < 0.001). The proportion of patients opting for TIV significantly decreased from 26.9% (14 patients) to 9.2% (6 patients) after 2014 (*P* = 0.023). The sex, clinical phenotypes, age at intervention, and time to treatment milestone (8.7 vs. 8.3 years; *P* = 0.680) showed no significant differences before and after 2014. Although there was no significant difference in age at onset (52.7 vs. 54.5 years; *P* = 0.659), patients who opted for TIV were consistently younger overall. In patients who did not opt for TIV, tracheostomy, or enteral nutrition, no significant differences in clinical characteristics, including age at onset, were observed between the two periods.

**TABLE 2:** Longitudinal clinical characteristics of patients by therapeutic intervention

Characteristic	No	Yes	*P* value	Before 2014	After 2014	*P* value
**TIV**						
Number of patients, *n*	97	20		14	6	0.023
Sex, *n* (Female/Male)	50/47	9/11	0.774	6/8	3/3	1.000
MSA‐C/ MSA‐P, *n*	60/37	15/5	0.390	10/4	5/1	1.000
Age at onset, y	63.6	53.2	<0.001	52.7	54.5	0.659
Age at death or intervention, y	71.7	63.4	<0.001	63.0	64.5	0.644
Survival time, y	7.4	16.9	<0.001	17.8	15.1	0.506
**Tracheostomy alone**						
Number of patients, *n*	70	55		42	13	<0.001
Sex, *n* (Female/Male)	36/34	26/29	0.779	19/23	7/6	0.822
MSA‐C/ MSA‐P, *n*	43/27	39/16	0.359	29/13	10/3	0.844
Age at onset, y	64.7	56.7	<0.001	58.3	51.5	<0.001
Age at death or intervention, y	71.6	64.1	<0.001	65.3	60.1	0.046
Survival time, y	6.9	13.1	<0.001	12.3	14.9	0.233
**Enteral nutrition**						
Number of patients, *n*	43	84		49	35	0.021
Gastrostomy tube, *n*	—	65		44	21	<0.001
Nasogastric tube, *n*	—	19		5	14	
Sex, *n* (Female/Male)	23/20	39/45	0.572	23/26	16/19	1.000
MSA‐C/ MSA‐P, *n*	35/8	49/35	0.016	30/19	19/16	0.681
Age at onset, y	64.6	59.7	0.006	57.4	62.9	0.011
Gastrostomy tube, y	—	57.8	<0.001	56.6	60.1	0.176
Nasogastric tube, y	—	66.4		64.5	67.1	0.523
Age at death or intervention, y	70.6	66.6	0.014	64.3	69.7	0.007
Gastrostomy tube, y	—	64.8	<0.001	63.5	67.3	0.109
Nasogastric tube, y	—	72.9		71.5	73.3	0.613
Survival time, y	5.7	10.2	<0.001	12.9	8.7	<0.001

Abbreviations: TIV, tracheostomy invasive ventilation; MSA‐cerebellar type; MSA‐P, MSA‐parkinsonian type; y, years (mean or median).

Treatment with tracheostomy alone was chosen for 55 patients (43.0%), and 70 patients (54.7%) died without tracheostomy. These patients were significantly younger at disease onset than those who did not undergo tracheostomy alone (56.7 vs. 64.7 years; *P* < 0.001). The proportion of patients undergoing tracheostomy significantly decreased from 65.6% (42 patients) to 21.3% (13 patients) after 2014 (*P* < 0.001). The sex, clinical phenotypes, and time to treatment milestone (7.0 vs. 7.5 years; *P* = 0.169) showed no significant differences after 2014. However, patients after 2014 had a significantly younger onset age compared to those before 2014 (51.5 vs. 58.3 years; *P* < 0.001).

Enteral nutrition was chosen for 84 patients (65.6%), and 43 patients (33.6%) died without enteral nutrition. These patients were significantly younger at disease onset than those who did not receive enteral nutrition (mean age, 59.7 vs. 64.6 years; *P* < 0.001). The proportion of patients opting for enteral nutrition decreased from 77% (42 patients) to 56% (35 patients) after 2014 (*P* = 0.021). The sex, clinical phenotypes, and time to treatment milestone (6.9 vs. 6.8 years; *P* = 0.854) showed no significant differences. However, onset age was significantly older after 2014 (57.4 vs. 62.9 years; *P* = 0.011). Compared with gastrostomy tubes, the use of nasogastric tubes increased after 2014, with their use continuing until the end of the observation period, reflecting patient preferences and clinical judgment.

## Discussion

This study offers insights into invasive treatments for MSA, including TIV, which are more common in Japan than Western countries, especially among ALS patients. This disparity may stem from various factors such as patient preferences,^1^ family influence,^7^ neurologist discretion,^8^ healthcare system differences, cultural influences, and religious beliefs.

Japan's unique healthcare system, cultural values, policies, and family structures influence the preference for invasive treatments like TIV compared to Western countries.^9^ In Japanese culture, there's a strong emphasis on familial responsibility and care, leading to a tendency towards life‐prolonging treatments for neurodegenerative diseases like MSA, even if they are invasive. The healthcare system, which provides comprehensive long‐term care, may also contribute to higher rates of such treatments.

The selection of invasive treatments in Japan has significantly decreased over the past two decades. This trend may reflect changing patient and family expectations, advances in palliative care, and the increasing adoption of international standards of care. Implementing Advanced Care Planning (ACP) principles could facilitate communication and shared decision‐making between patients and their caregivers. However, despite this decline in the selection of invasive treatments, the time to clinical milestones has remained stable during this period. This likely reflects MSA's uniform progression, the absence of disease‐modifying therapies, and consistent care practices.

The importance of ACP in MSA is increasingly recognized, yet its implementation remains insufficient. Only 37% of MSA patients have reportedly engaged in discussions about end‐of‐life care, with barriers attributed to insufficient education for healthcare providers and a lack of robust evidence.^10^ The findings of this study, which reveal trends and factors influencing invasive treatment choices, may serve as a foundation to support ACP implementation. Data‐driven explanations can help align care with patients’ preferences and overcome barriers to ACP adoption.

This analysis also highlights the significant influence of age on treatment preferences. Younger patients tend to opt for TIV, tracheostomy, and enteral nutrition, while older patients’ preferences evolved over time. This observation suggests that younger patients may lean towards more aggressive treatment options driven by a desire to prolong life. The trend continues even after 2014, with younger patients consistently opting for TIV and tracheostomy alone. Conversely, enteral nutrition is increasingly favored by older patients. Information on procedures like TIV is essential for choosing the right treatment. Understanding patient backgrounds is crucial in shaping policies.

Our findings indicate a notable decrease in the use of gastrostomy tubes, consistent with reports from Japan and the United States.^11^, ^12^ However, there's an increasing trend towards using less invasive options like nasogastric tubes. This shift underscores a preference for less invasive enteral nutrition options despite potential challenges like their impact on quality of life. This study shows a decline in invasive interventions over two decades, highlighting younger age's role in treatment choices and the need for tailored care strategies.

This study has several limitations. First, the data were collected from a single institution, which may limit the generalizability of our findings to broader populations or other regions. However, as a national referral center, our hospital captures a substantial portion of advanced MSA cases from this region. This minimizes the risk of selection bias while reflecting practices unique to our institution. Second, reliance on hospital medical records may introduce limitations in data accuracy and consistency due to variability in physicians’ documentation or potential observational bias. However, all cases had complete follow‐up data, and thorough chart reviews ensured detailed documentation.

Third, the study spanned 24 years, during which medical practices and patient preferences may have shifted. However, the consistent time to treatment milestones reflects the stable nature of MSA progression and the absence of disease‐modifying therapies. Finally, while the study is based in Japan, regional differences in treatment preferences within the country may exist, particularly between urban and rural areas. These differences could not be fully explored in this study due to sample size constraints. Future studies on these aspects would provide deeper insights.

## Author Roles

(1) Research project: A. Conception, B. Organization, C. Execution; (2) Statistical analysis: A. Design, B. Execution, C. Review and critique; (3) Manuscript preparation: A. Writing of the first draft, B. Review and critique.

K.N.: 1A, 1B, 1C, 2B, 3A.

K.S.: 3B.

N.F.: 3B.

## Disclosures

**Ethical Compliance Statement:** The study was approved by the institutional review board of Hyogo Chuo National Hospital (approval number: 20–05) and performed according to the Declaration of Helsinki. An opt‐out consent model was used to respect patient autonomy and confidentiality. We confirm that we have read the Journal's position on issues involved in ethical publication and affirm that this work is consistent with those guidelines.

**Funding Sources and Conflicts of Interest:** This work was supported by Grants‐in‐Aid from the Ministry of Health, Labour and Welfare, Japan (JPMH23FC1010). The authors declare that there are no conflicts of interest relevant to this work.

**Financial Disclosures for the Previous 12 Months:** All authors declare no financial or non‐financial competing interests.

## Data Availability

The data that support the findings of this study are available from the corresponding author upon reasonable request.
